# Safety, tolerability, pharmacokinetics, and pharmacodynamics of etrasimod in healthy Chinese adults: a randomized, double-blind, placebo-controlled dose-escalation phase 1 study

**DOI:** 10.3389/fphar.2025.1523339

**Published:** 2025-06-02

**Authors:** Fangfang Wang, Xiaoye Niu, Na Liu, Zhengying Zhu, Yun Lin, Lisa Ying, Haiyan Li

**Affiliations:** ^1^ Peking University Third Hospital, Beijing, China; ^2^ Everest Medicines, Shanghai, China

**Keywords:** etrasimod, sphingosine 1-phosphate receptor (S1PR), inflammatory bowel disease (IBD), lymphocytes, immune-mediated inflammatory diseases (IMID)

## Abstract

**Objectives:**

Etrasimod is an investigational, oral, once-daily, selective S1P_1,4,5_ receptor modulator in development for the treatment of immune-mediated inflammatory diseases. We present safety, tolerability, pharmacokinetic, and pharmacodynamic results of etrasimod treatment in healthy Chinese adults.

**Methods:**

In a Phase 1, randomized, double-blind, placebo-controlled, dose-escalation study, healthy Chinese adult subjects were randomly assigned to 3 cohorts. Cohorts 1 and 2 were given single-dose etrasimod, 1 mg or 2 mg, respectively, or placebo, followed by washout, then multiple-dose periods. Cohort 3 received multiple-dose etrasimod 2 mg or placebo, followed by titration to 3 mg or placebo. Cardiac monitoring included 24-h dynamic electrocardiogram, electrocardiogram monitoring, and 12-lead electrocardiogram. The primary endpoints were safety and tolerability, and secondary endpoints were pharmacokinetic and pharmacodynamic responses to etrasimod.

**Results:**

All treatment-emergent adverse events were Common Terminology Criteria for Adverse Events Grade 1 in severity, and all events were resolved without medical intervention. The most frequent event was sinus bradycardia (heart rate <50 bpm), and all these events were asymptomatic. No infections or infection-related events were reported. Pharmacokinetic and pharmacodynamic responses to etrasimod were consistent with previous studies in other populations. Etrasimod exposure increased at least dose proportionally for multiple doses and exhibited a half-life between 28.1 and 37.9 h. Etrasimod dose-dependently reduced lymphocyte counts, and these reductions were primarily seen in T naïve, T central memory, and T helper cells.

**Conclusion:**

Etrasimod was safe and well-tolerated in healthy Chinese subjects up to 3 mg in single and multiple-dose periods.

**Clinical Trial Registration:**

http://www.chinadrugtrials.org.cn, identifier: CTR20190003.

## Introduction

Immune-mediated inflammatory diseases (IMIDs) are a group of disabling chronic disorders characterized by dysregulated inflammatory signaling. IMIDs have a prevalence of 5%–7% in Western societies (El-Gabalawy et al., 2010). Ulcerative Colitis (UC) is an IMID affecting the gastrointestinal tract and colonic mucosa (inflammatory bowel diseases [IBDs]) (Brinkmann et al., 2010). The prevalence of IBDs is rising rapidly in Asian (Ng et al., 2018), with the current prevalence in China now exceeding 11.6/100,000 (Luo et al., 2015). This rising prevalence underscores the need to develop safe and effective therapies for Chinese populations.

UC therapies include anti-inflammatory or immunosuppressive agents, and biological agents such as infliximab, adalimumab, and other anti-tumor necrosis factor α treatments (Lee et al., 2018). However, many patients are therapy nonresponders or lose response over time (Singh et al., 2020). Additionally, many UC treatments require chronic parenteral administration or are associated with serious infections and malignancies (Agrawal et al., 2020). These considerations support continued development of novel therapies.

Etrasimod is an investigational, oral, once-daily, selective sphingosine 1-phosphate receptor subtype 1, 4, and 5 (S1P_1,4,5_) modulator, with no detectable activity on S1P_2,3_. Etrasimod regulates the recirculation of lymphocytes (Peyrin-Biroulet et al., 2017; Argollo et al., 2020). Etrasimod binds and internalizes the S1P_1_ receptor, sequestering lymphocyte subsets to lymphatic tissues, reversibly decreasing lymphocytes in peripheral blood and inflammatory sites. Etrasimod is a second generation S1P modulator, which aims to avoid serious adverse events (SAEs) associated with modulation of S1P_2_ and S1P_3_ (Jain and Bhatti, 2012; Calabresi et al., 2014; Argollo et al., 2020; D’Haens et al., 2023). The safety and efficacy of etrasimod has recently been demonstrated in patients with moderately to severely active UC (Sandborn et al., 2020; Sandborn et al., 2023; Vermeire et al., 2021). Etrasimod has been approved for the treatment of UC in the United States and Europe and is currently in Phase 2 and 3 development for multiple IMIDs (D’Haens et al., 2023). Etrasimod is rapidly absorbed and extensively metabolized through oxidation by several CYP enzymes (CYP2C8, 2C9, and 3A4), as well as dehydrogenation, sulfation, and glucuronidation. Etrasimod is primarily eliminated hepatically, as no renal elimination of unchanged etrasimod was detected in urine (Lee et al., 2018). While Phase 1 studies have shown favorable safety and pharmacokinetic (PK) properties of etrasimod (Lee et al., 2024), existing studies have limited representation of Asian populations (Sandborn et al., 2020; Sandborn et al., 2023; Lee et al., 2024) and safety data on Chinese individuals are limited. Furthermore, regulatory guidelines for New Drug Applications (NDAs) to the National Medical Products Administration of China (previously Chinese Food and Drug Administration) require the evaluation of safety, tolerability and PK profile within a Chinese population.

This Phase 1 study of etrasimod in healthy Chinese subjects was conducted to support the NDA submission of etrasimod in China. The objectives of the current study were to assess the safety, tolerability, PK and pharmacodynamic (PD) characteristics of etrasimod in healthy Chinese subjects.

## Methods

### Subjects and study design

This study (CTR20190,003) was a randomized, double-blind, placebo-controlled, dose-escalating Phase 1 clinical study in healthy Chinese subjects. Subjects were enrolled from Peking University Third Hospital, Beijing, China.

Subject eligibility required an age of 18 through 45 years, body weight ≥50 kg, and body mass index (BMI) of 19–24 kg/m^2^. Subjects had no clinically significant abnormalities according to physical examination, laboratory tests, and other required tests. Subjects were not allowed to take any prescriptions, over-the-counter medications, or herbs during the study. Effective contraceptive methods were required between signing informed consent to 3 months after last dose.

**FIGURE 1:**
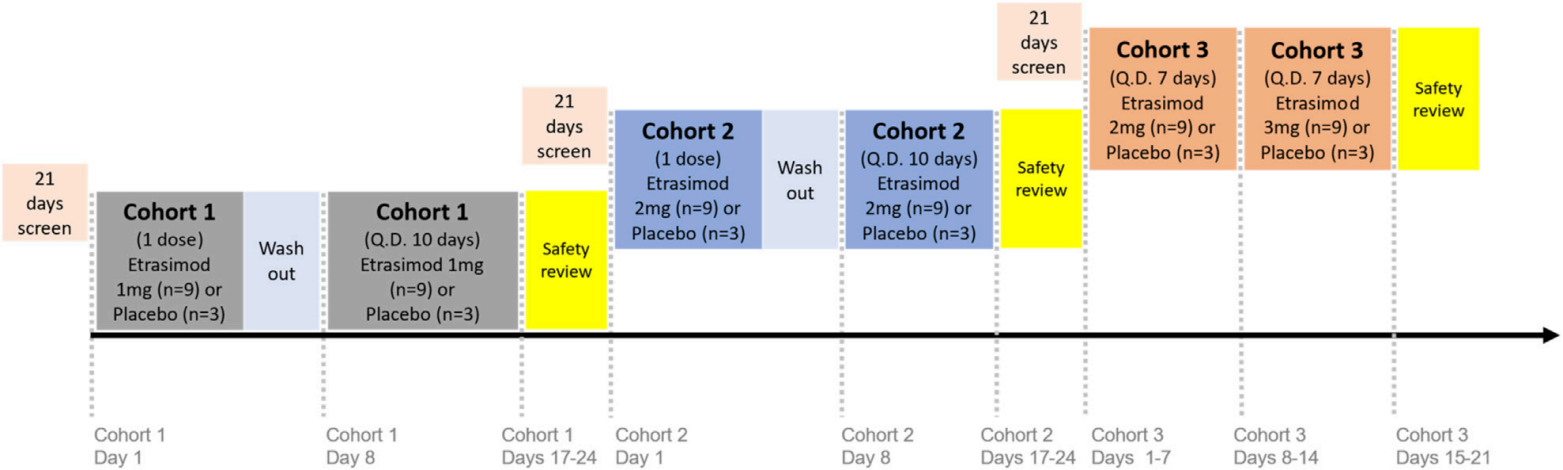
Study design. Subjects were randomized in a 3:1 ratio to receive once-daily oral etrasimod or placebo in either single-dose or multiple-dose periods: Cohort 1, etrasimod 1 mg or placebo; Cohort 2, etrasimod 2 mg or placebo; and Cohort 3, etrasimod 2 mg/3 mg or placebo. Cohorts 1 and 2 received assigned etrasimod or placebo during single-dose periods on Day 1, followed by 7-day washouts. Subjects then received 10 days of assigned etrasimod or placebo during multiple-dose periods occurring on Days 8 through 17. Cohort 3 received etrasimod 2 mg or placebo on Days 1–7, followed by etrasimod 3 mg or placebo on Days 8–14. Q.D., once daily.

Subjects were randomized 3:1 to receive once-daily oral etrasimod or placebo in single-dose or multiple-dose periods, with 12 subjects each in 3 cohorts (36 subjects total): Cohort 1, etrasimod 1 mg or placebo; Cohort 2, etrasimod 2 mg or placebo; and Cohort 3, etrasimod 2 mg/3 mg or placebo (Figure 1). Dosing was based on previous studies of etrasimod in healthy subjects or patients with UC (Sandborn et al., 2020; Lee et al., 2024). Cohorts 1 and 2 received assigned etrasimod or placebo during single -dose periods on Day 1, followed by 7-day washouts. Subjects then received 10 days of assigned etrasimod or placebo during multiple-dose periods occurring on Days 8 through 17.

In a previous single ascending dose (SAD) study, a pronounced heart rate reduction was observed at 3 mg of etrasimod (Lee et al., 2024). For this reason, it was decided that the 3 mg dose would be used with titration in etrasimod clinical development. In the current Phase 1 study, dose exploration for the highest dose (3 mg) was administered by titration. Cohort 3 received etrasimod 2 mg or placebo on Days 1–7, followed by etrasimod 3 mg or placebo on Days 8–14.

Prior to dose escalation, a safety review of the previous dose level was required. Safety review continued until discharge on Day 24 for Cohorts 1 and 2 and Day 21 for Cohort 3.

### Objectives and endpoints

The primary objective was to assess the safety and tolerability of etrasimod in healthy Chinese subjects. Adverse events (AEs) were recorded throughout the study. Additional safety assessments included clinical laboratory tests, physical and ophthalmologic examinations, pulmonary function tests, vital sign measurements, and cardiac measurements (12-lead electrocardiogram [ECG], ECG monitoring, and 24-h dynamic ECG). The clinical laboratory tests for safety monitoring included collection of blood samples for hematology. The testing of these samples was performed in real time to support the objective of safety monitoring during the study.

The secondary objectives of the study were to assess the PK properties and PD response to etrasimod in Chinese subjects. Blood samples for PK analyses were collected within 1 h predose, and 0.5, 1, 1.5, 2, 3, 3.5, 4, 4.5, 5, 6, 7, 8, 10, 12, 24, 48, 72, 96, 120, and 144 h postdose on Day 1 and Day 17 for Cohorts 1 and 2, and Day 14 for Cohort 3. Additional blood samples for PK analyses were taken for Cohorts 1 and 2 at 1 h predose on Days 15 and 16. Additional blood samples for PK analyses were taken for Cohort 3 within 1 h predose on Days 6, 12, and 13. On Day 7, samples were taken within 1 h predose and 0.5, 1, 1.5, 2, 3, 3.5, 4, 4.5, 5, 6, 7, 8, 10, 12, and 24 h postdose (predose on Day 8).

For PD responses, serial blood samples for absolute lymphocyte counts (ALCs) for Cohorts 1 and 2 were taken within 1 h predose and at 1, 2, 4, 8, 12, 24, 48, 72, 96, 120, and 144 h postdose on Day 1, within 1 h predose on Day 8, and 4 h postdose on Days 8, 9, 11, 13, 15, 17, 19, and 24. Samples for ALC for Cohort 3 were taken within 1 h predose on Day 1 and 4 h postdose on Days 1, 3, 5, 7, 8, 9, 11, 13, 14, 16, and 21. Blood samples for immunophenotyping were taken from Cohort 2 within 1 h predose and 4 and 24 h postdose on Day 1,within 1 h predose on Day 8, and 4 h postdose on Day 17. Absolute peripheral blood lymphocyte counts for PD evaluation were determined by direct immunofluorescent staining with fluorochrome-conjugated monoclonal antibodies in whole blood and analysis on the FACSCanto™ II flow cytometer (Becton Dickinson, Franklin Lakes, NJ, United States).

### Bioanalytical methods

Concentrations of etrasimod in human plasma were determined according to an established protocol using a validated liquid chromatography tandem mass spectrometry (LC-MS/MS) assay. The validated analytical range was 0.250–100 ng/mL using 100 µL of plasma, where 0.250 was the lower limit of quantitation. Quantitation of peak area ratios of the analyte and internal standard were determined using weighted linear regression analysis (1/concentration^2^). The dilution quality control level was 750 ng/mL. Following incurred sample reanalysis, 99.2% of samples met acceptance criteria. The inter-run precision (percentage coefficient of variation) was 1.8%–3.8%. The inter-run accuracy (percentage of relative error) was −0.1% to −1.5%.

### Statistical methods

Subjects who received placebo in any cohort were analyzed as a single group. For safety data, treatment-emergent adverse events (TEAEs) were summarized as the number and percentage of subjects and TEAEs, and heart rate (HR) was summarized as a mean change from baseline in beats per minute (bpm) by mean and standard deviation (SD). Log-transformed PK concentration and parameter data were summarized by geometric mean and geometric coefficient of variation. The PK parameters, area under the concentration-time curve (AUC) and maximum observed plasma concentration (C_max_), of etrasimod were assessed for dose proportionality during the multiple-dose period using the power model (Gough et al., 1995). The PK parameters of etrasimod in the single dose period and multiple doses period were calculated based on the actual sampling time using non-compartmental analysis methods within Phoenix WinNonlin Version 8.1. PD responses were calculated as percent change from baseline and summarized using mean and SD for ALCs and peripheral blood immune cell subtype counts.

Sample sizes in each cohort were selected based on recommendations from the “Technical Guidelines for clinical pharmacokinetic study of chemical drugs” issued by the National Medical Products Administration in March 2005, which requires 8–12 subjects per dose group. Accordingly, 12 healthy subjects were enrolled in each dose group. The sample size was not based on statistical power calculations for the primary objective, which was to assess the safety and tolerability of etrasimod. No formal statistical comparisons were planned and conducted. All data processing, summarization, and analyses were performed with SAS Version 9.4 or higher.

### Study ethics and patient consent

This study was done in compliance with the Declaration of Helsinki, the International Council for Harmonisation Good Clinical Practice Guidelines, and relevant laws and local regulations. The protocol was approved by the Peking University Third Hospital Institutional Review Board. All subjects provided written informed consent.

## Results

### Subjects

Thirty-six subjects were randomized and entered the study. All subjects completed the study except for 1 subject, who discontinued treatment from Cohort 3 because of a TEAE [atrioventricular block (AVB) second degree Mobitz Type I] after a single dose of etrasimod 2 mg (described in the safety section).

Demographics were generally similar between the treatment groups. In total, 24 (66.7%) subjects were male and 12 (33.3%) were female, with similarly-distributed group sex ratios. Mean BMI values were similar between groups. For etrasimod 1 mg, etrasimod 2 mg, etrasimod 2 mg/3 mg, and placebo groups, respectively, mean BMI (±SD) was 22.19 ± 1.619 kg/m^2^, 21.57 ± 1.051 kg/m^2^, 22.56 ± 1.190 kg/m^2^, and 21.89 ± 1.501 kg/m^2^.

### Safety

#### Adverse events

The proportions of TEAEs were similar across all groups (Table 1). All TEAEs were mild [Common Terminology Criteria for Adverse Events (CTCAE) Grade 1] and resolved without treatment. The most common TEAEs by preferred term (PT) in etrasimod exposed subjects were sinus bradycardia, followed by alanine aminotransferase increased, white blood cells urine positive, and blood triglycerides increased. Twenty-one subjects experienced drug-related TEAEs (as determined by the investigator) during the study: 5 (55.6%), 7 (77.8%), 8 (88.9%), and 1 (11.1%) subjects in the etrasimod 1 mg, etrasimod 2 mg, and etrasimod 2 mg/3 mg, and the placebo groups, respectively.

**TABLE 1:** Summary of common treatment-emergent adverse events by preferred term (≥2 subjects in either combined etrasimod group or placebo group).

System organ class	Etrasimod 1 mg (N = 9) n (%)	Etrasimod 2 mg (N = 9) n (%)	Etrasimod 2 mg/3 mg (N = 9) n (%)	Placebo (N = 9) n (%)
Preferred term^a^				
Subjects with at least one TEAE	7 (77.8)	8 (88.9)	9 (100.0)	8 (88.9)
Blood and lymphatic system disorders				
Leukopenia	0	2 (22.2)	1 (11.1)	0
Cardiac disorders				
Atrioventricular block first degree	1 (11.1)	1 (11.1)	1 (11.1)	0
Atrioventricular block second degree	0	0	2 (22.2)	0
Sinus bradycardia	2 (22.2)	3 (33.3)	6 (66.7)	1 (11.1)
Investigations				
Alanine aminotransferase increased	0	3 (33.3)	2 (22.2)	0
Aspartate aminotransferase increased	0	2 (22.2)	0	0
Blood triglycerides increased	2 (22.2)	1 (11.1)	1 (11.1)	0
Blood urine present	2 (22.2)	0	1 (11.1)	1 (11.1)
Electrocardiogram QRS complex prolonged	0	2 (22.2)	0	0
Gamma-glutamyltransferase increased	0	1 (11.1)	1 (11.1)	0
Neutrophil percentage increased	0	0	2 (22.2)	0
Protein urine present	0	0	2 (22.2)	0
Red blood cells urine positive	0	0	2 (22.2)	1 (11.1)
Urinary sediment present	1 (11.1)	0	1 (11.1)	0
White blood cell count decreased	2 (22.2)	0	1 (11.1)	0
White blood cells urine positive	2 (22.2)	1 (11.1)	2 (22.2)	1 (11.1)
Vascular disorders				
Hypotension	0	2 (22.2)	1 (11.1)	2 (22.2)

Note: TEAEs, presented in the table included TEAEs, during the single-dose periods and the multiple-dose periods; TEAEs, in the single-dose periods were events with a start date on or after the date of first study drug dose and before the first study drug dose in the multiple-dose period. TEAEs, in the multiple-dose periods were non-serious events with a start date on or after the date of first study drug dose in the multiple-dose periods and within 7 days after the last dose, or serious events with a start date on or after the date of first study drug dose in the multiple-dose periods and within 30 days after the last dose.

^a^ Preferred terms with ≥2 subjects reported to have the TEAE, in either combined etrasimod treatment groups or placebo group are listed. For maximum CTCAE, grade, subjects with multiple AEs, within a particular category were counted once under the category of their highest-grade AE, within that category.

AE, adverse events; CTCAE, common terminology criteria for adverse events; N, number of subjects; TEAE, treatment-emergent adverse event.

Heart rate reduction and AV conduction delay are known cardiac effects after the first dose of etrasimod (Lee et al., 2024). In this study, 11 out of 27 subjects in the combined etrasimod groups, and 1 out of 9 subjects in the placebo group were reported to have sinus bradycardia (HR < 50 bpm) as a TEAE, mainly on the first dosing day or upon dose escalation. All events were detected by ECG or Holter monitoring. Only one subject in the etrasimod group had an HR < 45 bpm on the first dosing day. Three subjects in the etrasimod groups experienced AVB first degree, with 1 subject in each dose group, and 2 of 3 subjects exhibited AVB first degree before their first dose. Two subjects in the etrasimod 2 mg/3 mg group had AVB second degree Mobitz Type I during the 2 mg period. Among these 2 subjects, one subject had a single episode of AVB second degree Mobitz Type I, which was judged by the investigator as not related to the study drug; the event was captured by Holter monitoring 8 h postdose on Day 1 and lasted for 2 s with an HR of 58 bpm. This subject remained asymptomatic and completed all subsequent dosing with no further atrioventricular conduction delay detected. The second subject exhibited AVB second degree Mobitz Type I at 5 h postdose on Day 1 with HR of 50 bpm, as measured with ECG. All subsequent ECG measurements from 6 h postdose through 24 h postdose showed no further atrioventricular conduction delay. The investigator considered the event to be probably related to etrasimod and discontinued the subject’s participation as a precaution, according to the prespecified discontinuation criteria. All bradycardia and AV conduction delay events were Grade 1 in severity, asymptomatic, transient, and resolved without medical intervention. No action was taken with the study drug, except for the precautionary discontinuation of the participant mentioned above.

There were no deaths, SAEs, or important medical adverse events reported during the study. No dose-interrupting TEAEs were reported. One subject had a TEAE resulting in study drug discontinuation (as described above).

#### Heart rate

**FIGURE 2:**
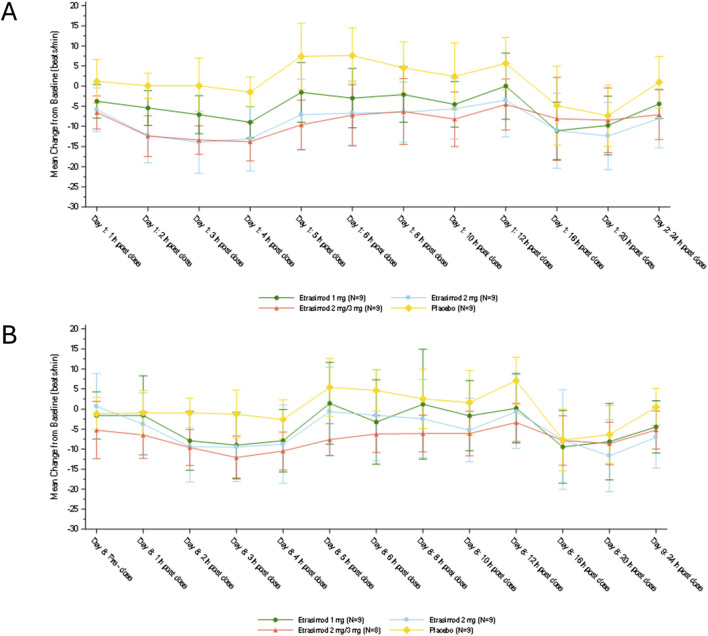
Change from baseline in heart rate on **(A)** Day 1 and **(B)** Day 8. On both Day 1 and Day 8, 12-lead electrocardiogram was used to measure heart rate at 1-h predose and 1, 2, 3, 4, 5, 6, 8, 10, 12, and 24-h post-dose, and Holter monitoring was used to measure heart rate at 16 and 20 h postdose. Plots show mean ± standard deviation.

The mean changes from baseline in HR on Days 1 and 8 are shown in Figure 2. On Day 1, the first HR decline, as measured by ECG, in all 3 etrasimod groups started at approximately 1-h postdose and the maximum decline in HR occurred at 3–4 h postdose. On Day 1, up to 8 h postdose, the placebo-corrected least-square mean changes from baseline in HR to nadir [± standard error (SE)], were −6.5 ± 1.64 bpm, −11.0 ± 1.64 bpm, and −12.2 ± 1.64 bpm for the etrasimod 1 mg group, etrasimod 2 mg group, and the etrasimod 2 mg/3 mg group (2 mg period), respectively. A second decline in HR, as measured by Holter monitoring, occurred during night sleep between 16 to 20 h postdose and was comparable between all 4 treatment groups. These similar reductions suggest the etrasimod first dose-effect did not influence the lowest HR during sleep. HR reductions and associated TEAEs were asymptomatic. On Day 8, up to 8 h postdose, the placebo-corrected least-square mean changes from baseline to HR nadir (±SE) were −6.7 ± 1.99 bpm, −6.9 ± 1.99 bpm, and −8.4 ± 2.05 bpm for the etrasimod 1 mg group, etrasimod 2 mg group, and the etrasimod 2 mg/3 mg group (3 mg period), respectively. The changes in HR from baseline to nadir on Day 8 (first dose date of the multiple-dose periods for 1 mg and 2 mg, and the first titration date of 3 mg) were similar between the 3 etrasimod groups.

No clinically significant changes in mean PR interval, QRS, QT, and QTc interval from baseline were observed for all treatment groups during ECG monitoring.

#### Other safety parameters of special interest

Vital signs, physical exams, and ophthalmological exams were stable throughout the study. No clinically significant changes from baseline in pulmonary function and ophthalmologic measures were observed. No subject experienced an increase of more than 3 × ULN for liver enzymes, and enzyme abnormalities were asymptomatic, returned to normal without any intervention, and all events were mild. Real-time monitoring of hematology showed a dose-related mean reduction of lymphocytes following etrasimod treatment. The lowest mean (SD) reductions from baseline in lymphocyte counts were −0.66 (0.45), −1.06 (0.27), −1.15 (0.45), and −0.15 (0.16) ×10^9^/L, for etrasimod 1 mg, etrasimod 2 mg, etrasimod 2 mg/3 mg, and placebo groups respectively. The mean lymphocyte counts returned to >80% of baseline on discharge day (7 days after last dose) for all dose levels. No infection related TEAEs were reported.

### Pharmacokinetics

**FIGURE 3:**
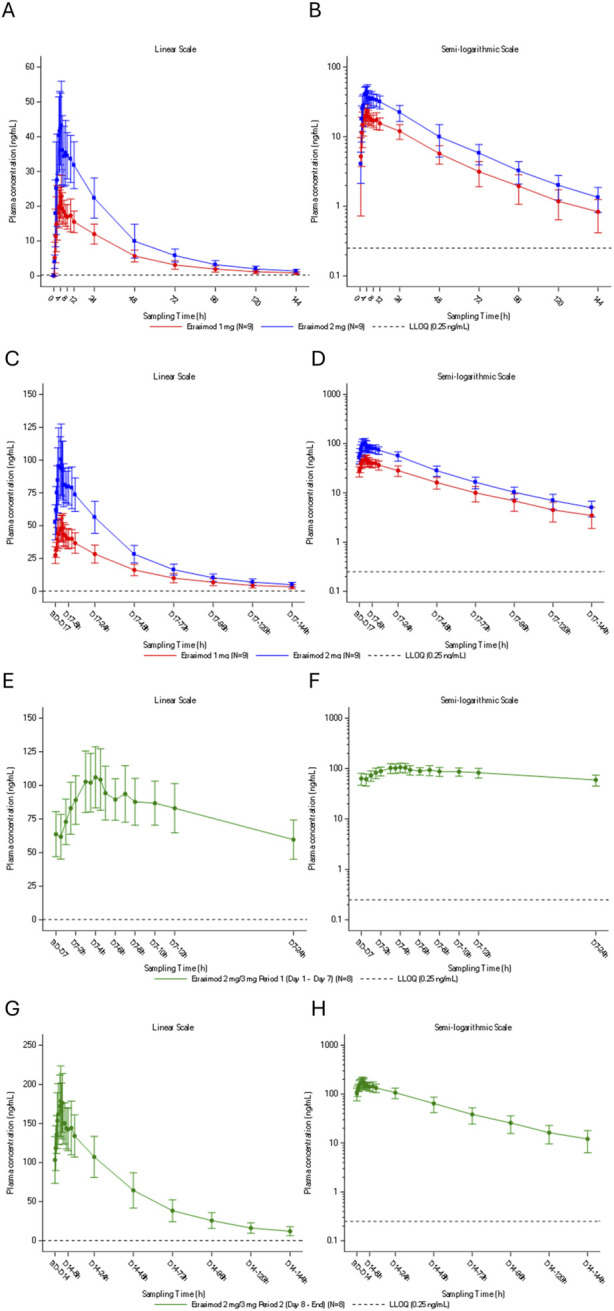
Plasma concentration-time profile of etrasimod following **(A,B)** single oral doses of etrasimod 1 mg or etrasimod 2 mg, **(C,D)** multiple once-daily oral doses of etrasimod 1 mg or etrasimod 2 mg, **(E,F)** multiple once-daily oral doses of etrasimod 2 mg for the etrasimod 2 mg/3 mg group, **(G,H)** multiple once-daily oral doses of etrasimod 3 mg for the etrasimod 2 mg/3 mg group. Data are presented as arithmetic mean and are shown on a linear scale **(A,C,E,G)** and a semi-logarithmic scale **(B,D,F,H)**. h, hours; LLOQ, lower limit of quantification; PK, pharmacokinetics.

Plasma concentration-time profiles of etrasimod for both single-dose and multiple dose treatment are summarized in Figure 3.

#### Single dose PK parameters

Following a single oral administration under fasting conditions, the median time of maximum observed plasma concentration (T_max_) was 3.5 h and 4.0 h in the etrasimod 1 mg and 2 mg groups, respectively. The geometric mean of apparent plasma terminal elimination half-life (t_½_) was 30.1 h and 28.1 h in the etrasimod 1 mg and 2 mg groups, respectively. T_max_, t_½_ and the geometric means of ${\text{MRT}}_{0-\infty }$ , V_z_/F, CL/F, and λz were also comparable between single-dose groups. C_max_ was 23.5 ng/mL and 44.6 ng/mL for etrasimod 1 mg and etrasimod 2 mg, respectively. During the single-dose periods, the plasma exposure of etrasimod was dose proportional for C_max_, AUC_0-t_, AUC_0-24_, and ${\text{AUC}}_{0-\infty }$ (Table 2).

**TABLE 2:** Etrasimod single dose pharmacokinetic parameters.

Parameter	Etrasimod 1 mg (N = 9) Geometric mean (CV%)	Etrasimod 2 mg (N = 9) Geometric mean (CV%)
C_max_ (ng/mL)	23.5 (27.0)	44.6 (23.6)
AUC_0-t_ (h*ng/mL)	775 (23.0)	1,460 (23.7)
AUC_0-24_ (h*ng/mL)	360 (20.6)	694 (19.9)
${\text{AUC}}_{0-\infty }$ (h*ng/mL)	810 (24.0)	1,510 (24.1)
AUC_%Extrap_ (%)	4.02 (47.4)	3.35 (30.5)
T_max_ (h)^a^	3.5 (1.5, 4.5)	4.0 (3.0, 8.0)
t_½_ (h)	30.1 (13.1)	28.1 (6.6)
${\text{MRT}}_{0-\infty }$ (h)	42.7 (15.7)	40.6 (9.4)
V_z_/F (L)	53.5 (19.9)	53.5 (21.6)
CL/F (L/h)	1.23 (24.0)	1.32 (24.1)
λz (L/h)	0.0231 (13.1)	0.0247 (6.6)

Notes: Geometric mean (CV%) data are presented. Descriptive statistics (except N, min and max) were calculated only if ≥ 3 individual data were provided. ${\text{AUC}}_{0-\infty }$ with more than 30% from the extrapolated values were excluded in summary statistics and could not be included in calculation of the related PK parameters (V_z_/F, CL/F and ${\text{MRT}}_{0-\infty }$ ).

^a^ Median (min-max) was presented.

λz, apparent terminal elimination rate constant; AUC_0-t_, area under the plasma concentration-time curve from time zero to t; AUC_0-24_, area under the plasma concentration-time curve from time 0–24 h postdose; ${\text{AUC}}_{0-\infty }$ , area under the plasma concentration-time curve from time zero to infinity; AUC_%Extrap_, percentage of ${\text{AUC}}_{0-\infty }$ due to extrapolation from T_last_ to infinity; CL/F, apparent total plasma clearance following oral administration; C_max_, maximum observed plasma concentration; CV, coefficient of variation; MRT_0-t_, mean residence time from time 0 to the last measurable concentration; ${\text{MRT}}_{0-\infty }$ , mean residence time from time 0 extrapolated to infinity; N, number of subjects; PK, pharmacokinetic; SD, standard deviation; t_1/2_, apparent plasma terminal elimination half-life; T_max_; time of maximum observed plasma concentration; V_z_/F; apparent volume of distribution during the terminal phase.

#### Multiple dose PK parameters

Following daily oral administration under fasting conditions, the multiple-dose periods median T_max_ for etrasimod was 3.5–4.5 h. The geometric mean of t_½_ for etrasimod ranged from 33.2 to 37.9 h. Based on similar trough concentrations, steady state was achieved on Days 15–17 in the etrasimod 1 and 2 mg groups, Days 6–7 in the 2 mg/3 mg group (2 mg period), and Days 12–14 in the 2 mg/3 mg group (3 mg period). The multiple-dose etrasimod plasma exposure accumulation ratio of 1 mg and 2 mg was 2.13 to 2.70-fold higher compared to single-dose administration (Table 3). During multiple-dose periods, the plasma exposure of etrasimod was dose proportional from 1 mg to 2 mg and from 2 mg to 3 mg for the maximum observed plasma concentration at steady state (C_max,ss_), the average plasma concentration at steady state (C_avg_), and the AUC_(0-24)_. The plasma exposure was slightly more than dose -proportional from 1 mg to 3 mg (Table 4).

**TABLE 3:** Etrasimod multiple dose pharmacokinetic parameters.

Parameter	Etrasimod 1 mg (N = 9) Geometric mean (CV%)	Etrasimod 2 mg (N = 9) Geometric mean (CV%)	Etrasimod 2 mg/3 mg (2 mg period) (N = 8) Geometric mean (CV%)	Etrasimod 2 mg/3 mg (3 mg period) (N = 8) Geometric mean (CV%)
λz (1/h)	0.0183 (8.7)	0.0209 (8.3)	NA	0.0189 (15.5)
t_½_ (h)	37.9 (8.7)	33.2 (8.3)	NA	36.8 (15.5)
T_max_ (h)^a^	4.5 (3.0, 6.0)	3.5 (2.0, 7.0)	4.0 (3.0, 4.5)	3.5 (2.0, 6.0)
C_max,ss_ (ng/mL)	49.9 (22.2)	101 (28.8)	107 (23.6)	179 (23.1)
AUC_0-τ_ (h*ng/mL)	870 (20.3)	1720 (21.8)	1870 (22.9)	3,170 (20.6)
AUC_0-t_ (h*ng/mL)^b^	2090 (25.0)	3,820 (24.6)	1860 (22.9)	7,770 (28.5)
C_avg_ (ng/mL)	36.2 (20.3)	71.7 (21.8)	78.0 (21.2)	132 (20.6)
DF (%)	63 (25.8)	71.0 (15.4)	NA	60.4 (21.3)
CL_ss_/F (L/h)	1.15 (20.3)	1.16 (21.8)	1.07 (22.9)	0.948 (20.6)
V_z,ss_/F (L)	62.9 (15.1)	55.6 (18.3)	NA	50.3 (19.4)
${\text{MRT}}_{0-\infty }$ (h)	49.6 (16.1)	43.7 (9.0)	NA	50.7 (15.7)
Rac(C_max_)	2.13 (22.5)	2.27 (13.0)	NA	NA
Rac(AUC_0-24_)	2.42 (16.0)	2.48 (15.1)	NA	NA
Rac(AUC_0-t_)	2.70 (14.4)	2.62 (20.5)	NA	NA
TCP	1.07 (11.3)	1.14 (18.0)	NA	NA
Day 6 C_trough_ (ng/mL)	NA	NA	58.3 (21.1)	NA
Day 7 C_trough_ (ng/mL)	NA	NA	61.9 (25.9)	NA
Day 12 C_trough_ (ng/mL)	NA	NA	NA	94.1 (25.9)
Day 13 C_trough_ (ng/mL)	NA	NA	NA	99.8 (23.8)
Day 14 C_trough_ (ng/mL)	NA	NA	NA	99.9 (27.5)
Day 15 C_trough_ (ng/mL)	25.6 (21.8)	48.1 (24.4)	NA	NA
Day 16 C_trough_ (ng/mL)	25.7 (25.2)	52.1 (27.4)	NA	NA
Day 17 C_trough_ (ng/mL)	26.9 (23.4)	50.8 (29.8)	NA	NA

^a^ Median (min-max) was presented.

^b^ 0 to 24 h postdose for the 2 mg dose-escalation period in 2 mg/3 mg group, 0–144 h for the 1 mg group, the 2 mg group, and the 3 mg-dose period of the 2 mg/3 mg group.

λz, terminal elimination rate constant; AUC_0-t_, area under the plasma concentration-time curve from time zero to t; AUC_0-τ_, area under the plasma concentration-time curve from time 0 to the end of the dosing interval, where τ (tau) is 24 h; Css,max, maximum observed plasma concentration at steady state; Cavg, average plasma concentration at steady state calculated as AUC_0-τ_/τ; CL_ss_/F, apparent steady-state plasma clearance following oral administration, calculated as Dose/AUC_0- τ_; C_trough_, trough (predose) plasma concentration; CV, coefficient of variation; D, day; DF, degree of fluctuation (%) calculated as 100 × (C_max_-C_min_)/C_avg_; h, hour; ${\text{MRT}}_{0-\infty }$ , mean residence time from time 0 extrapolated to infinity; N, number of subjects; NA, not applicable; Rac(C_max_), accumulation calculated as Day 17 Cmax/Day 1 Cmax; Rac(AUC_0-t_), accumulation ratio based on area under the plasma concentration-time curve from time 0 to the time of last measurable concentration; Rac(AUC_0-24_), accumulation ratio based on area under the plasma concentration-time curve from time 0–24 h postdose; t_1/2_, apparent plasma terminal elimination half-life; T_max_, time of maximum observed plasma concentration; TCP, temporal change parameter calculated as Day 17 AUC_0-τ_/Day 1 ${\text{AUC}}_{0-\infty }$ ; V_z,ss_/F, apparent volume of distribution at steady state, calculated as Dose/λZ × AUC_0-τ_.

**TABLE 4:** Power model for dose-proportionality analysis of pharmacokinetic parameters during the multiple-dose period–pharmacokinetic set.

PK parameters (unit)	Slope	90% CI for the slope		Pooled geometric CV%
		Lower	Upper	
C_ss,max_ (ng/mL)	1.2346	1.1387	1.3304	24.84
C_avg_ (ng/mL)	1.2543	1.1563	1.3524	21.78
AUC_0-t_ (h*ng/mL)	1.1517	0.9486	1.3547	27.83
AUC_0-τ_ (h*ng/mL)	1.2449	1.1339	1.3558	21.73

Abbreviations: AUC_0-t_, area under the plasma concentration-time curve from time zero to time t; AUC_0-τ_, area under the plasma concentration-time curve from time zero to the end of the dosing interval; C_avg_, average plasma concentration at steady state calculated as AUC_0-τ_/τ; C_ss,max_, maximum observed plasma concentration at steady state; CI, confidential interval; CV, coefficient of variation; PK, pharmacokinetic.

### Pharmacodynamics

Etrasimod induced a rapid, dose-dependent, and reversible reduction in lymphocytes. Peripheral blood ALCs were evaluated before and after etrasimod treatment.

#### Single dose PD

In the single-dose periods (Days 1–7), ALC declined modestly within 2 h following dosing. In the etrasimod 1 mg, 2 mg and placebo groups, the mean percentage changes from baseline to ALC nadir were −33.19%, −46.17%, and −15.76%, respectively. The mean times to reach ALC nadir were observed 36.89, 28.89, and 52.56 h postdose, respectively. ALC returned to baseline levels 5 days after the single dose in both etrasimod groups.

#### Multiple dose PD

In the multiple-dose periods, the mean percentage changes in lymphocyte counts from baseline to nadir were −45.68%, −65.93%, −73.01%, and −13.87% in the etrasimod 1 mg, 2 mg, 2 mg/3 mg, and placebo groups, respectively. The associated mean times to lymphocyte count nadir ranged from 4 to 7 days postdose, with values of 156.00, 177.34, 124.00, and 105.33 h, respectively. ALC gradually returned to near baseline within 7 days after last dose (Day 24 for Cohorts 1 and 2, Day 21 for Cohort 3).

#### PK-PD relationship

**FIGURE 4:**
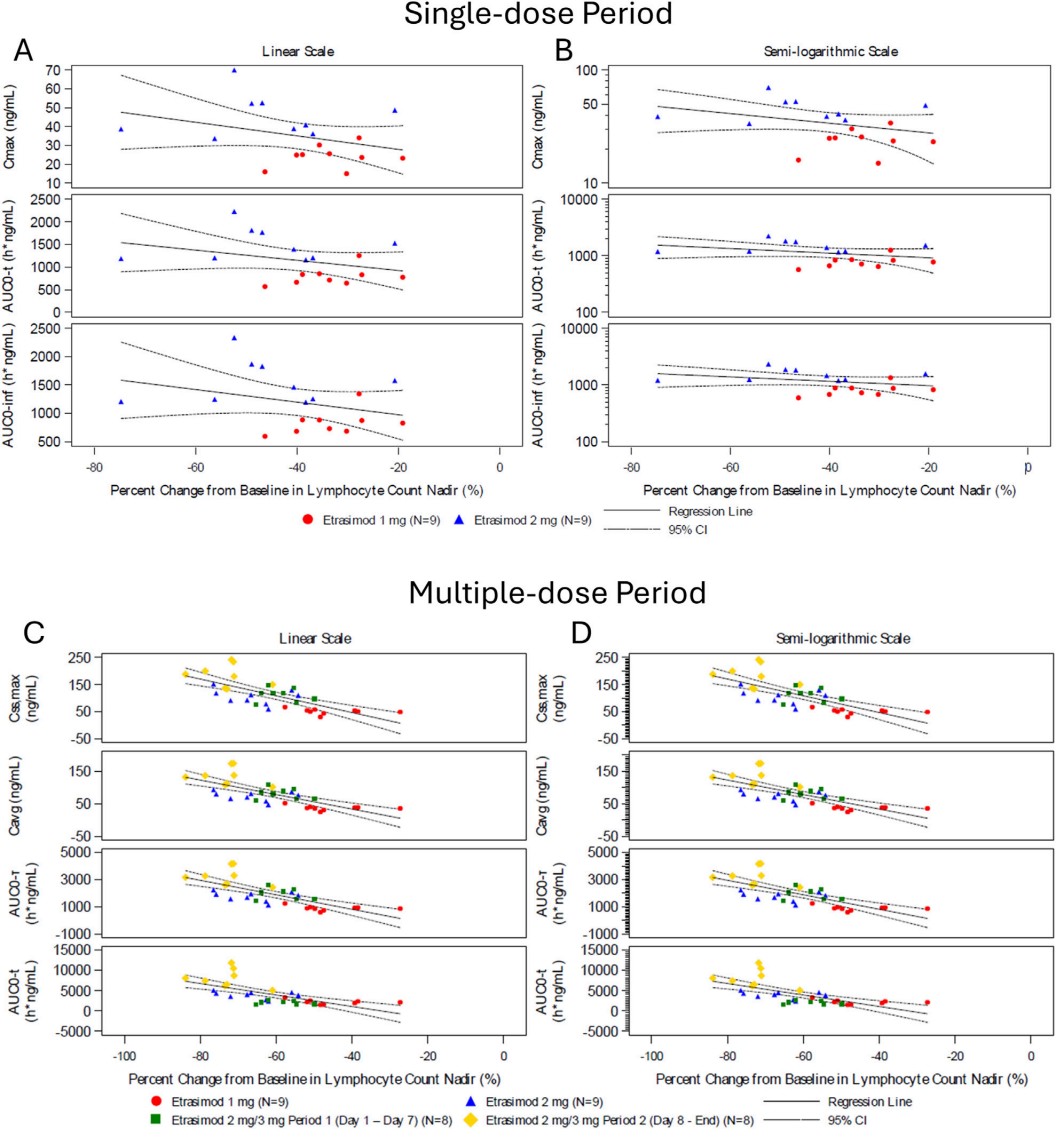
Percent change from baseline in lymphocyte count nadir and pharmacokinetic parameter during the single-dose and multiple-dose period. Scatter plots of percent change in lymphocyte count nadir and PK parameter values following etrasimod doses of 1 mg (red circles, n = 9), 2 mg (blue triangle, n = 9), 2/3 mg period 1 (green square, n = 8), and 2/3 mg period 2 (yellow diamond, n = 8). Data are presented in the single dose **(A,B)** and multiple-dose **(C,D)** period on a linear scale **(A,C)** and semi-logarithmic scale **(B,D)**. AUC_0-inf_, area under the plasma concentration-time curve from time zero to infinity; AUC_0-t_, area under the plasma concentration-time curve from time zero to time t; AUC_0-τ_, area under the concentration-time curve over the dosing interval; where tau (τ) = 24 h; C_avg_, average plasma concentration at steady state calculated as AUC_0-τ_/τ; C_,max_, maximum observed plasma concentration; C_ss,max_, maximum observed plasma concentration at steady state; CI, confidential interval.

During the single-dose and multiple-dose periods, the etrasimod 3 mg group exhibited a greater percentage decrease in lymphocyte count nadir compared to the 2 mg and 1 mg groups. This reduction was associated with higher C_max_, AUC_0-inf_, and AUC_0-t_ (Figure 4). This association suggests a dose-dependent reduction in peripheral lymphocyte counts with increasing etrasimod exposure.

#### Immune cell subtypes

Immune cell subtypes were measured in subjects from Cohort 2 on Day 1 and Day 17. The reduction from baseline was primarily seen in T naïve, T helper, and T central memory cells, and to a lesser extent, T cytotoxic and T effector memory cells (Table 5).

**TABLE 5:** Percent change from baseline in peripheral immune cell subtypes.

	Mean % change from baseline on day 17 (SD)	
Cell type	Etrasimod 2 mg once daily (N = 9)	Placebo (N = 3)
T-helper cells (CD3+/CD4+)	−80.50 (8.87)	18.32 (11.31)
T-cytotoxic cells (CD3+/CD8+)	−60.86 (13.81)	7.72 (14.70)
T-naïve cells (CCR7+/CD45RA+/CD3+)	−90.99 (4.47)	2.52 (14.61)
T-effector memory cells (CCR7-/CD45R-/CD3+)	−38.58 (14.09)	52.72 (7.80)
T-central memory cells (CCR7+/CD45R-/CD3+)	−69.24 (13.50)	−10.29 (2.62)
B Cells (CD19^+^)	−80.39 (6.49)	39.62 (41.14)
Natural killer cells (CD3-/CD56+)	−20.73 (30.50)	−17.08 (30.42)

Abbreviations: CCR, chemokine receptors; CD, cluster of differentiation; CD45RA, long isoform of CD45; SD, standard deviation.

## Discussion

This was the first study evaluating the safety, tolerability, PK, and PD of a range of single and multiple doses of etrasimod in healthy Chinese subjects. The results of this Phase 1 study show that oral etrasimod was safe and well tolerated up to 3 mg, including multiple doses, in healthy Chinese subjects.

Heart rate reduction and AV conduction delay are known effects within the S1P receptor modulator class. All TEAEs reported were CTCAE Grade 1 in severity and resolved without treatment. There were no severe TEAEs or SAEs reported during the study. The most frequent TEAE was sinus bradycardia due to HR reduction. Only 1 subject reported a TEAE of bradycardia with HR < 45 bpm on the first dosing day. Two subjects reported 2 TEAEs of AVB second degree Mobitz Type I. In this study, cardiac events occurred within hours of the first dose, attenuated after repeated dosing, and were mild in severity, transient and asymptomatic. All events resolved without medical intervention. The heart rate lowing was dose-dependent, with the largest reduction occurring at 3-4 h postdose from the predose baseline. These findings were consistent with those from previous global SAD and MAD studies (Lee et al., 2024).

The on-target HR-slowing effects of S1P_1_ modulators required titration to effective dose in the labeling of some drugs in this class (Sandborn et al. 2020). Due to the favorable safety profile of etrasimod, patients were started at the target dose of 2 mg without titration in the etrasimod pivotal studies, ELEVATE UC52 and ELEVATE UC12 (Sandborn et al., 2023). Here, healthy Chinese subjects were administered etrasimod 1 mg and 2 mg safely without titration. In Cohort 3, the 7-day titration with 2 mg attenuated any further HR-slowing effects of etrasimod 3 mg. No significant change in pulmonary function, ophthalmologic measures, or liver function was observed in the study. No infections or infection related TEAEs were reported in this study.

In Chinese healthy volunteers, etrasimod absorption was rapid: the median time to maximum plasma concentration ranged from 3.5 to 4.5 h after single and multiple doses of etrasimod, for all dose levels. Geometric mean t_½_ ranged from 28.1 to 37.9 h, similar to the previously reported half-life for etrasimod (Lee et al., 2024). For the multiple-dose periods, etrasimod plasma exposure increased dose-dependently from 1 mg to 2 mg and from 2 mg to 3 mg for C_max,ss_, C_avg_, and AUC_(0-τ)_, and more than dose-proportionally from 1 mg to 3 mg.

Etrasimod is a small molecule S1P_1_ modulator, which partially and reversibly sequesters lymphocytes in the lymphoid organs, causing a subsequent reduction in a subset of circulating lymphocytes (Sandborn et al., 2023). Here, an etrasimod dose-dependent reduction in lymphocyte count was observed, similar in magnitude to previous studies (Schreiber et al., 2017; Lee et al., 2024). During the single-dose and multiple-dose periods, the etrasimod 3 mg group showed a greater decrease in the lymphocyte count nadir compared to the 2 mg and 1 mg groups. This reduction was linked to higher C_max_, AUC_0-inf_, and AUC_0-t_ values, indicating a dose-dependent decline in peripheral lymphocyte counts with increased etrasimod exposure. This reduction was followed by a rapid recovery and ALCs returned towards baseline 7 days after the last dose. The favorable PK/PD profile supports the continued clinical development of etrasimod. In etrasimod Phase 1 studies and UC Phase 3 pivotal studies, ALCs returned to near baseline within 7 days following drug discontinuation and to the normal range in approximately 80% of subjects at the earliest follow-up visit, 2 weeks after cessation of longterm etrasimod treatment (Lee et al., 2024; Sandborn et al., 2023). The current findings confirm a fast wash-out period for etrasimod in healthy Chinese subjects, distinguishing etrasimod from other S1P modulators (Taylor et al., 2022). Lymphocyte reductions were further investigated to identify the affected cellular subtypes. Etrasimod 2 mg primarily reduced peripheral blood counts of T naïve, T central memory, and T helper cells, with smaller reductions in T cytotoxic and T effector memory cells. These observations are consistent with the expected retention of CCR7+ cells in secondary lymphocyte organs.

This study should be interpreted in the context of some limitations. Consistent with other Phase 1 studies, small numbers of healthy participants were evaluated and a short study duration precluded assessment of long-term safety and immune effects of lymphocyte depletion. Evaluation of the long-term effects of etrasimod is in progress in a Phase 3 study enrolling subjects from mainland China, Taiwan, and South Korea (NCT04176588; CTR20192294). In the present study, due to the titration design of the etrasimod 3 mg multiple dose period from a 2 mg multiple dose period, a single-dose PK profile for etrasimod 3 mg could not be obtained.

In conclusion, etrasimod was safe and well tolerated by healthy Chinese subjects. The safety profile and PK and PD effects were consistent with previous etrasimod studies in other populations. In addition, titration attenuated any further HR-lowering effects of 3 mg etrasimod. These data suggest oral, once daily etrasimod in doses up to 3 mg is safe for continued evaluation of etrasimod for the treatment of IMIDs.

## Data Availability

The raw data supporting the conclusions of this article will be made available by the authors, without undue reservation.
